# Evaluation of Glial and Neuronal Blood Biomarkers Compared With Clinical Decision Rules in Assessing the Need for Computed Tomography in Patients With Mild Traumatic Brain Injury

**DOI:** 10.1001/jamanetworkopen.2022.1302

**Published:** 2022-03-14

**Authors:** Linda Papa, Jay G. Ladde, John F. O’Brien, Josef G. Thundiyil, James Tesar, Stephen Leech, David D. Cassidy, Jesus Roa, Christopher Hunter, Susan Miller, Sara Baker, Gary A. Parrish, Jillian Davison, Christine Van Dillen, George A. Ralls, Joshua Briscoe, Jay L. Falk, Kurt Weber, Philip A. Giordano

**Affiliations:** 1 Department of Emergency Medicine, Orlando Regional Medical Center, Orlando, Florida

## Abstract

**Importance:**

In 2018, the combination of glial fibrillary acidic protein (GFAP) and ubiquitin C-terminal hydrolase (UCH-L1) levels became the first US Food and Drug Administration–approved blood test to detect intracranial lesions after mild to moderate traumatic brain injury (MTBI). How this blood test compares with validated clinical decision rules remains unknown.

**Objectives:**

To compare the performance of GFAP and UCH-L1 levels vs 3 validated clinical decision rules for detecting traumatic intracranial lesions on computed tomography (CT) in patients with MTBI and to evaluate combining biomarkers with clinical decision rules.

**Design, Setting, and Participants:**

This prospective cohort study from a level I trauma center enrolled adults with suspected MTBI presenting within 4 hours of injury. The clinical decision rules included the Canadian CT Head Rule (CCHR), New Orleans Criteria (NOC), and National Emergency X-Radiography Utilization Study II (NEXUS II) criteria. Emergency physicians prospectively completed data forms for each clinical decision rule before the patients’ CT scans. Blood samples for measuring GFAP and UCH-L1 levels were drawn, but laboratory personnel were blinded to clinical results. Of 2274 potential patients screened, 697 met eligibility criteria, 320 declined to participate, and 377 were enrolled. Data were collected from March 16, 2010, to March 5, 2014, and analyzed on August 11, 2021.

**Main Outcomes and Measures:**

The presence of acute traumatic intracranial lesions on head CT scan (positive CT finding).

**Results:**

Among enrolled patients, 349 (93%) had a CT scan performed and were included in the analysis. The mean (SD) age was 40 (16) years; 230 patients (66%) were men, 314 (90%) had a Glasgow Coma Scale score of 15, and 23 (7%) had positive CT findings. For the CCHR, sensitivity was 100% (95% CI, 82%-100%), specificity was 33% (95% CI, 28%-39%), and negative predictive value (NPV) was 100% (95% CI, 96%-100%). For the NOC, sensitivity was 100% (95% CI, 82%-100%), specificity was 16% (95% CI, 12%-20%), and NPV was 100% (95% CI, 91%-100%). For NEXUS II, sensitivity was 83% (95% CI, 60%-94%), specificity was 52% (95% CI, 47%-58%), and NPV was 98% (95% CI, 94%-99%). For GFAP and UCH-L1 levels combined with cutoffs at 67 and 189 pg/mL, respectively, sensitivity was 100% (95% CI, 82%-100%), specificity was 25% (95% CI, 20%-30%), and NPV was 100%; with cutoffs at 30 and 327 pg/mL, respectively, sensitivity was 91% (95% CI, 70%-98%), specificity was 20% (95% CI, 16%-24%), and NPV was 97%. The area under the receiver operating characteristic curve (AUROC) for GFAP alone was 0.83; for GFAP plus NEXUS II, 0.83; for GFAP plus NOC, 0.85; and for GFAP plus CCHR, 0.88. The AUROC for UCH-L1 alone was 0.72; for UCH-L1 plus NEXUS II, 0.77; for UCH-L1 plus NOC, 0.77; and for UCH-L1 plus CCHR, 0.79. The GFAP biomarker alone (without UCH-L1) contributed the most improvement to the clinical decision rules.

**Conclusions and Relevance:**

In this cohort study, the CCHR, the NOC, and GFAP plus UCH-L1 biomarkers had equally high sensitivities, and the CCHR had the highest specificity. However, using different cutoff values reduced both sensitivity and specificity of GFAP plus UCH-L1. Use of GFAP significantly improved the performance of the clinical decision rules, independently of UCH-L1. Together, the CCHR and GFAP had the highest diagnostic performance.

## Key Points

**Question:**

How does the diagnostic performance of serum glial fibrillary acidic protein (GFAP) and neuronal ubiquitin C-terminal hydrolase (UCH-L1) biomarkers compare with that of validated clinical decision rules for detecting intracranial lesions on computed tomography (CT) of the head in patients with mild traumatic brain injury, and is a combination of biomarkers and clinical decision rules associated with improved performance?

**Findings:**

In this cohort of 349 patients with mild traumatic brain injury, the Canadian CT Head Rule (CCHR), the New Orleans Criteria (NOC), and GFAP plus UCH-L1 had 100% sensitivity for detecting lesions on CT, but the CCHR had the highest specificity (33%), followed by GFAP plus UCH-L1 (25%) and the NOC (16%). The combination of GFAP level and the CCHR yielded the highest diagnostic performance.

**Meaning:**

These findings suggest that although GFAP plus UCH-L1 and the clinical decision rules performed similarly in detecting intracranial lesions on CT scans, the diagnostic performance was improved when biomarkers were combined with rules, specifically GFAP with the CCHR.

## Teaser

This cohort study compares the performance of validated clinical decision rules vs levels of US Food and Drug Administration–approved biomarkers glial fibrillary acidic protein and ubiquitin C-terminal hydrolase in detecting traumatic intracranial injuries on computed tomography in patients presenting to a level I trauma center with suspected mild traumatic brain injury.

## Introduction

Traumatic intracranial injuries requiring prompt acute intervention occur in less than 10% of patients with mild traumatic brain injury (MTBI),^1^ and intracranial injuries leading to death or requiring neurosurgical intervention occur in less than 1%.^1^ Computed tomographic (CT) scan of the brain is the diagnostic standard for acute evaluation of suspected traumatic intracranial injuries in the emergency department (ED), and CT has considerably improved diagnostic capabilities and reduced hospital admissions.^2^ Still, efforts are ongoing to reduce use of CT to prevent unnecessary radiation exposure while maintaining quality of care and moderating cost.^3,4,5^ Independently derived and validated clinical decision rules, including the Canadian CT Head Rule (CCHR), New Orleans Criteria (NOC), and National Emergency X-Radiography Utilization Study II (NEXUS II) criteria, allow for selective use of CT in patients with suspected MTBI^6,7,8,9^ and have shown reductions in rates of CT scans by 20% to 30%.^10,11^

Glial and neuronal serum biomarkers glial fibrillary acidic protein (GFAP) and ubiquitin C-terminal hydrolase (UCH-L1) have been evaluated in several studies to detect acute traumatic intracranial lesions on CT scan after an MTBI in adults^12,13,14,15,16,17,18,19^ and children, with sensitivities ranging from 94% to 100%.^19,20,21,22^ They are both detectable in serum within 1 hour of injury.^15,19^ Levels of GFAP peak at 20 hours after injury and slowly decline for 72 hours but are still detectable at 7 days. Levels of UCH-L1 peak at 8 hours and decline rapidly for 48 hours.^15,19^ In 2018, GFAP and UCH-L1 were approved by the US Food and Drug Administration (FDA) for clinical use in adult patients with MTBI to help determine the need for CT scan within 12 hours of injury.^23^ Such a blood test offers an objective measure that could complement clinical decision-making.

The potential impact of these biomarkers on MTBI management will remain unknown unless their performance can be directly compared with current clinical decision guidelines. Such a comparison would help clinicians weigh risks and benefits before introducing them into clinical practice. The primary objectives of this study were (1) to prospectively compare the performance of validated clinical decision rules (the CCHR, the NOC, and NEXUS II) vs the FDA-approved biomarkers GFAP and UCH-L1 in detecting traumatic intracranial injuries on CT in patients presenting to a level I trauma center with suspected MTBI and (2) to evaluate whether biomarkers would improve the diagnostic accuracy of the clinical decision rules. Secondary objectives included assessing physician comfort level with each rule and physician attitudes toward the potential application of a TBI blood test to their clinical practice.

## Methods

### Study Population

This prospective cohort study enrolled a convenience sample of adult patients with MTBI presenting to the ED of a level I trauma center in Orlando, Florida, within 4 hours of injury from March 16, 2010, to March 5, 2014. This study was approved by the institutional review board of Orlando Regional Medical Center, and written informed consent was obtained from each patient and/or their legal authorized representative before enrollment. This study followed the Strengthening the Reporting of Observational Studies in Epidemiology (STROBE) reporting guideline for cohort studies.

Eligibility owing to suspected MTBI was determined by the treating physician based on the history of blunt head trauma followed by a change in sensorium such as loss of consciousness, amnesia, or disorientation and presenting to the ED within 4 hours of injury with a Glasgow Coma Scale score of 13 to 15. Eligibility was also prospectively verified by the research team before enrollment. Head CT scans were performed at the discretion of the treating physician. Patients were excluded if they (1) were younger than 18 years; (2) had no history of trauma as their primary event (eg, syncope or seizure); (3) had known dementia, chronic psychosis, or active central nervous system pathology; (4) were pregnant; (5) were incarcerated; or (6) had a systolic blood pressure of less than 100 mm Hg.

### Study Procedures

All initial patient assessments were made by board-certified emergency medicine physicians trained by a formal 1-hour session on evaluating patient eligibility for the study. The training session and study protocol did not alter or influence physician practice in any way. Application of clinical decision rules was at the discretion of the treating physician. After the initial screening, a meticulous secondary assessment was conducted by the research team in the ED to ensure tha each patient met inclusion criteria and verified any exclusion. All prehospital and ED records were reviewed, and patients, families, and witnesses (if available) were questioned.

After the clinical examination and before the CT, the clinical decision rule assessment was performed prospectively by the treating clinician. Treating physician comfort level (5-point Likert scale of very comfortable, comfortable, neutral, uncomfortable, and very uncomfortable) with each decision rule was also collected prospectively for each patient at the time of assessment.

Blood samples were obtained from each patient with MTBI within 4 hours of the reported time of injury. For each blood draw, a single vial of approximately 5 mL of blood was collected and placed in clot tubes with a serum separator and allowed to clot at room temperature. The blood was centrifuged within 30 minutes and the serum was placed in bar-coded aliquot containers and stored in a freezer at −70°C until it was transported to a central laboratory. There, the samples were analyzed in batches using sandwich enzyme-linked immunosorbent assays (ELISAs) for GFAP and UCH-L1. Laboratory personnel running the samples were blinded to the clinical data. After assessment and treatment in the ED, patients were either discharged home or admitted to the hospital based on the severity of their injuries, and patient management was not altered by the study.

### Outcome Measures

The primary outcome for patients was presence of an acute traumatic intracranial lesion visualized on a CT scan, including such findings as extra-axial lesions (eg, epidural, subdural, and subarachnoid hemorrhage) or intra-axial lesions (eg, contusions, intraparenchymal hemorrhage, cerebral edema, traumatic axonal injury, and midline shift of intracranial contents), as well as any signs of brain herniation or pneumocephalus. The ordering pattern for CT scans at this trauma center is such that most patients with blunt head injury and subsequent symptoms have a head CT scan performed as part of usual care.^15,19^ The CT examinations were interpreted by board-certified radiologists and neuroradiologists who recorded the location, extent, and type of brain injury. They were blinded to the contents of the data collection sheet but were aware of the patient’s clinical history.

### Biomarker Analysis

Serum GFAP and UCH-L1 levels were measured in duplicate for each sample using a validated ELISA platform (Banyan Biomarkers, Inc). For the GFAP assay, the lower limit of quantification was 30 pg/mL (to convert to ng/mL, multiply by 0.001), and the upper limit of quantification was 50 000 pg/mL. The limit of detection was 8 pg/mL. For the UCH-L1 assay, the lower limit of quantification was 100 pg/mL (to convert to ng/mL, multiply by 0.001), and the upper limit of quantification was 9000 pg/mL. The limit of detection was 45 pg/mL. Any samples yielding a signal greater than the quantification or calibrator range were diluted and assayed a second time.

### Statistical Analysis

Data were analyzed on August 11, 2021. Descriptive statistics with means and proportions were used. Biomarker levels were treated as continuous data and expressed as medians with IQRs. Data were assessed for equality of variance and distribution, and logarithmic transformations were conducted accordingly. Performance of the biomarkers along with the 3 clinical decision rules was compared using logistic regression. Receiver operating characteristic (ROC) curves were created to explore the ability of the biomarkers to detect intracranial lesions on CT scan. Estimates of the area under the ROC curves (AUROC) were obtained (AUROC of 0.50 indicates no discrimination; AUROC of 1.00 indicates a perfect diagnostic test). Classification performance was assessed by sensitivity, specificity, positive (PPV) and negative (NPV) predictive values, and likelihood ratios with 95% CIs.

Two sets of prespecified biomarker cutoff values were used in the analysis based on (1) cutoff values derived in previous studies^14,21^ that used ROC curve analysis to maximize sensitivity for identification of CT lesions (67 pg/mL for GFAP and 189 pg/mL for UCH-L1) and (2) cutoff values derived from a recent validation study sponsored by Banyan Biomarkers, Inc^18^ (22 pg/mL for GFAP and 327 pg/mL for UCH-L1), that informed the FDA approval. Given that the limit of detection for GFAP was 8 pg/mL and the lower limit of quantification was 30 pg/mL for this study, 30 pg/mL was used as a cutoff value instead of 22 pg/mL.

A negative test result was defined as both marker levels below their cutoff value, whereas a positive test result was defined as 1 or both biomarker levels above their cutoff value. Analyses were performed using the statistical software package SPSS, version 27.0 (IBM Corporation).

Physician comfort level with each of the rules was recorded for each enrolled participant and presented descriptively using proportions. In addition, we calculated the false-positive rate of CT scans to indicate how many unnecessary CT scans would have been ordered based on the results of the biomarkers and clinical decision rules. Two-sided *P* < .05 indicated statistical significance.

### Sample Size Calculation

Based on our previous work with biomarkers and CT head rules, a sample size was calculated a priori.^15,24^ A sample of 22 from the group with positive CT findings and 220 from the group with negative CT findings (total of 240 patients) achieves an 80% power to detect a difference of an AUROC of 0.15 between a diagnostic test with an AUROC of 0.80 (biomarkers) and another diagnostic test (clinical decision rules) with an AUROC of 0.65 using a 2-sided *z* test at a significance level of *P* < .05.

## Results

**Figure 1.**
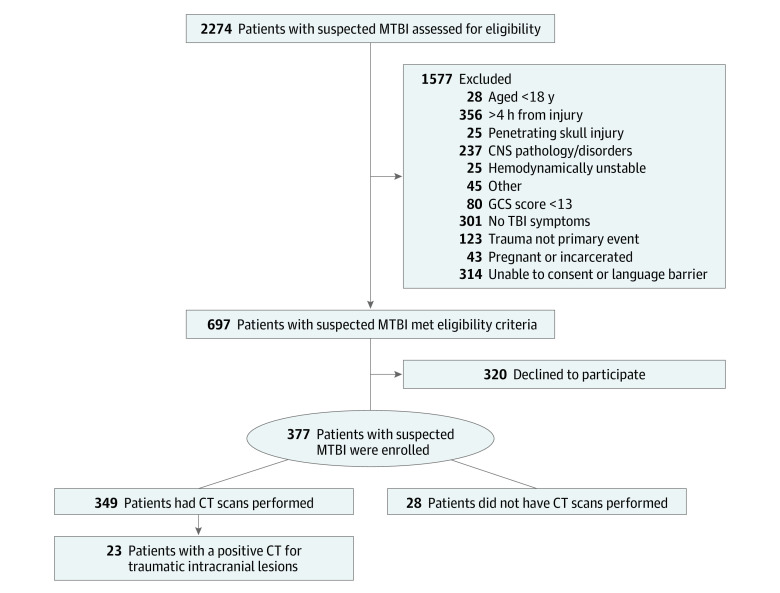
Flow Diagram of Screened and Enrolled Patients CNS indicates central nervous system; CT, computed tomography; GCS, Glasgow Coma Scale; and MTBI, mild traumatic brain injury.

Of 2274 patients with suspected MTBI screened in the ED, 697 (31%) met eligibility criteria and 320 (46%) declined to participate (Figure 1). A total of 377 eligible patients with suspected MTBI enrolled, and 349 (93%) who had CT scans performed in the ED were included in the analysis. The mean (SD) age of enrolled patients was 40 (16) years; 230 (66%) were men and 119 (34%) were women. Racial and ethnic data were collected during hospital registration. Reporting race and ethnicity in this study was mandated by the US National Institutes of Health, consistent with the Inclusion of Women, Minorities, and Children policy. Describing racial and ethnic composition in research is important for generalizability of results. The racial and ethnic distribution included 6 (2%) Asian patients, 62 (18%) Black patients, 70 (20%) Hispanic patients, and 202 (58%) White patients (Table 1). Among eligible patients not enrolled, patient characteristics were similar to those of enrolled patients in age, sex, and race and ethnicity. The 3 most common injury mechanisms were motor vehicle crashes (162 [46%]), falls (71 [20%]), and motorcycle crashes (44 [13%]). A total of 314 patients (90%) presented with a Glasgow Coma Scale score of 15; 118 (34%) were admitted to the hospital; and 23 (7%) had evidence of a traumatic intracranial lesion on CT. A comparison of characteristics of patients with suspected MTBI with and without intracranial lesions on CT scan are presented in Table 1. The mean time from injury to blood sample was 182 (95% CI, 177-186) minutes.

**Table 1.** Characteristics of the 349 Patients With Suspected MTBI and CT Scans Performed in the Emergency Department

Characteristic	Patient group^a^			*P* value
	CT with negative findings (n = 326)	CT with positive findings (n = 23)	All (n = 349)	
Age, mean (SD), y	40 (16)	47 (15)	40 (16)	.03
Sex				
Women	114 (35)	5 (22)	119 (34)	.26
Men	212 (65)	18 (78)	230 (66)	
Race and ethnicity				
Asian	4 (1)	2 (9)	6 (2)	.08
Black	60 (18)	2 (9)	62 (18)	
Hispanic	67 (21)	3 (13)	70 (20)	
White	186 (57)	16 (70)	202 (58)	
Other, unknown, or not reported^b^	9 (3)	0	9 (3)	
GCS score				
13	2 (1)	0	2 (1)	.08
14	27 (8)	6 (26)	33 (9)	
15	297 (91)	17 (74)	314 (90)	
Mechanism of injury				
Motor vehicle collision	156 (48)	6 (26)	162 (46)	.22
Motorcycle collision	40 (12)	4 (17)	44 (13)	
Other motorized vehicle	5 (2)	1 (4)	6 (2)	
Bicycle struck by vehicle	12 (4)	2 (9)	14 (4)	
Fall from bicycle	5 (2)	0	5 (1)	
Pedestrian struck	13 (4)	1 (4)	14 (4)	
Fall	62 (19)	9 (39)	71 (20)	
Sports injury	4 (1)	0	4 (1)	
Assault	16 (5)	0	16 (5)	
Other	13 (4)	0	13 (4)	
Mode of transport ambulance	300 (92)	22 (96)	322 (92)	.42
Admitted to hospital	95 (29)	23 (100)	118 (34)	<.001
Loss of consciousness	213 (65)	22 (96)	235 (67)	.002
Amnesia	105 (32)	12 (52)	117 (34)	.07
CT lesions^c^				
Basal skull fracture	0	2 (9)	2 (1)	NA
Subarachnoid hemorrhage	0	13 (57)	13 (4)	
Subdural hematoma	0	10 (43)	10 (3)	
Contusion or parenchymal hemorrhage	0	7 (30)	7 (2)	
Epidural hematoma	0	1 (4)	1 (<1)	
Traumatic axonal injury	0	3 (13)	3 (1)	
Skull fracture	0	9 (39)	9 (3)	

Abbreviations: CT, computed tomography; GCS, Glasgow Coma Scale; MTBI, mild traumatic brain injury; NA, not applicable.

^a^ Unless otherwise indicated, data are expressed as number (%) of patients. Percentages are rounded and may not equal 100%.

^b^ Includes multiracial patients.

^c^ Some patients have more than 1 characteristic.

The proportions of variables from each clinical decision rule found in enrolled participants are presented in eTable 1 in the Supplement. The most frequently cited variable of the CCHR was dangerous mechanism (194 [56%]); of the NOC, trauma above the clavicles (188 [54%]); and of the NEXUS II, scalp hematoma (97 [28%]).

The classification performance of the 3 clinical decision rules for identifying traumatic intracranial lesions on CT is presented in Table 2. Both CCHR and NOC had sensitivities of 100% (95% CI, 82%-100%) and NEXUS II had a sensitivity of 83% (95% CI, 60%-94%). NEXUS II had the highest specificity at 52% (95% CI, 47%-58%), followed by CCHR at 33% (95% CI, 28%-39%) and NOC at 16% (95% CI, 12%-20%). The NPV was 100% for both CCHR (95% CI, 96%-100%) and NOC (95% CI, 91%-100%) and 98% (95% CI, 94%-99%) for NEXUS II.

**Table 2.** Classification Performance of 3 Validated Clinical Decision Rules for Determining the Need for Head CT Scan in Patients With Suspected MTBI

Result of assessment	Clinical decision rule		
	CCHR	NOC	NEXUS II
Rule finding, No. of patients			
Positive, injury/no injury	23/218	23/274	19/156
Negative, injury/no injury	0/108	0/52	4/170
Sensitivity, % (95% CI)	100 (82-100)	100 (82-100)	83 (60-94)
Specificity, % (95% CI)	33 (28-39)	16 (12-20)	52 (47-58)
PPV, % (95% CI)	10 (6-14)	8 (5-12)	11 (7-17)
NPV, % (95% CI)	100 (96-100)	100 (91-100)	98 (94-99)
Likelihood ratio (95% CI)	1.50 (1.39-1.61)	1.19 (1.13-1.25)	1.73 (1.39-2.15)
Unnecessary CTs, No. (%)^a^	218/349 (62)	274/349 (79)	156/349 (45)

Abbreviations: CCHR, Canadian CT Head Rule; CT, computed tomography; MTBI, mild traumatic brain injury; NEXUS II, National Emergency X-Radiography Utilization Study II; NOC, New Orleans Criteria; NPV, negative predictive value; PPV, positive predictive value.

^a^ Defined by false-positive results (positive test result and negative CT finding).

The classification performance of the 2 serum biomarkers, individually and in combination, were compared using cutoff values of 67 pg/mL for GFAP and 189 pg/mL for UCH-L1 and a second set of cutoff values of 30 pg/mL for GFAP and 327 pg/mL for UCH-L1 (Table 3). The sensitivity of GFAP and UCH-L1 in combination was higher with the first set of prespecified values (67 and 189 pg/mL, respectively) than with the second set (30 and 327 pg/mL, respectively) at 100% (95% CI, 82%-100%) vs 91% (95% CI, 70%-98%), respectively; specificity for the 2 sets of values were 25% (95% CI, 20%-30%) vs 20% (95% CI, 16%-24%), respectively. Because GFAP with a cutoff of 67 pg/mL plus UCH-L1 with a cutoff of 189 pg/mL outperformed GFAP with a cutoff of 30 pg/mL plus UCH-L1 with a cutoff of 327 pg/mL, the former set was used in all subsequent analyses for the dichotomized GFAP-UCH-L1 combination.

**Table 3.** Classification Performance of Serum Biomarkers GFAP and UCH-L1 Alone and in Combination Based on Prespecified Cutoff Values

Result of assessment	Biomarker		
	GFAP	UCH-L1	GFAP and UCH-L1
**GFAP level cutoff 67 pg/mL, UCH-L1 level cutoff 189 pg/mL** ^a^			
Biomarker finding, No. of patients			
Positive, injury/no injury	20/113	22/232	23/245
Negative, injury/no injury	3/213	1/94	0/81
Sensitivity, % (95% CI)	87 (65-97)	96 (76-100)	100 (82-100)
Specificity, % (95% CI)	65 (60-70)	29 (24-34)	25 (20-30)
PPV, % (95% CI)	15 (10-23)	9 (6-13)	9 (6-13)
NPV, % (95% CI)	99 (96-100)	99 (93-100)	100 (94-100)
Likelihood ratio (95% CI)	2.50 (2.02-3.12)	1.34 (1.20-1.50)	1.33 (1.25-1.42)
Unnecessary CT, No. (%)^b^	113/349 (32)	232/349 (66)	245/349 (70)
**GFAP level cutoff 30 pg/mL, UCH-L1 level cutoff 327 pg/mL^c^**			
Biomarker finding, No. of patients			
Positive, injury/no injury	21/219	18/183	21/262
Negative, injury/no injury	2/107	5/143	2/64
Sensitivity, % (95% CI)	91 (70-98)	78 (56-92)	91 (70-98)
Specificity, % (95% CI)	33 (28-38)	44 (38-49)	20 (16-24)
PPV, % (95% CI)	9 (6-13)	9 (6-14)	7 (5-11)
NPV, % (95% CI)	98 (93-100)	97 (92-99)	97 (89-99)
Likelihood ratio (95% CI)	1.36 (1.17-1.57)	1.39 (1.10-1.76)	1.14 (0.99-1.30)
Unnecessary CT, No. (%)^b^	219/349 (63)	183/349 (52)	262/349 (75)

Abbreviations: CT, computed tomography; GFAP, glial fibrillary acidic protein; NPV, negative predictive value; PPV, positive predictive value; UCH-L1, ubiquitin C-terminal hydrolase.

^a^ Based on Papa et al.^14,21^

^b^ Defined by false-positive results (positive test result and negative CT finding).

^c^ Based on Bazarian et al.^18^

To weigh the sensitivity and specificity together, the AUROC was calculated for the different prespecified biomarker cutoffs and clinical decision rules (eTable 2 in the Supplement). Concentration of GFAP alone with a cutoff of 67 pg/mL had the highest AUROC (0.76 [95% CI, 0.67-0.85]), followed by CCHR (AUROC, 0.67 [95% CI, 0.58-0.75]) and NEXUS II (AUROC, 0.67 [95% CI, 0.57-0.78]). The lowest AUROCs were for NOC (0.58 [95% CI, 0.47-0.69]) and GFAP with a cutoff of 30 pg/mL plus UCH-L1 with a cutoff of 327 pg/mL (0.56 [95% CI, 0.44-0.67]).

**Figure 2.**
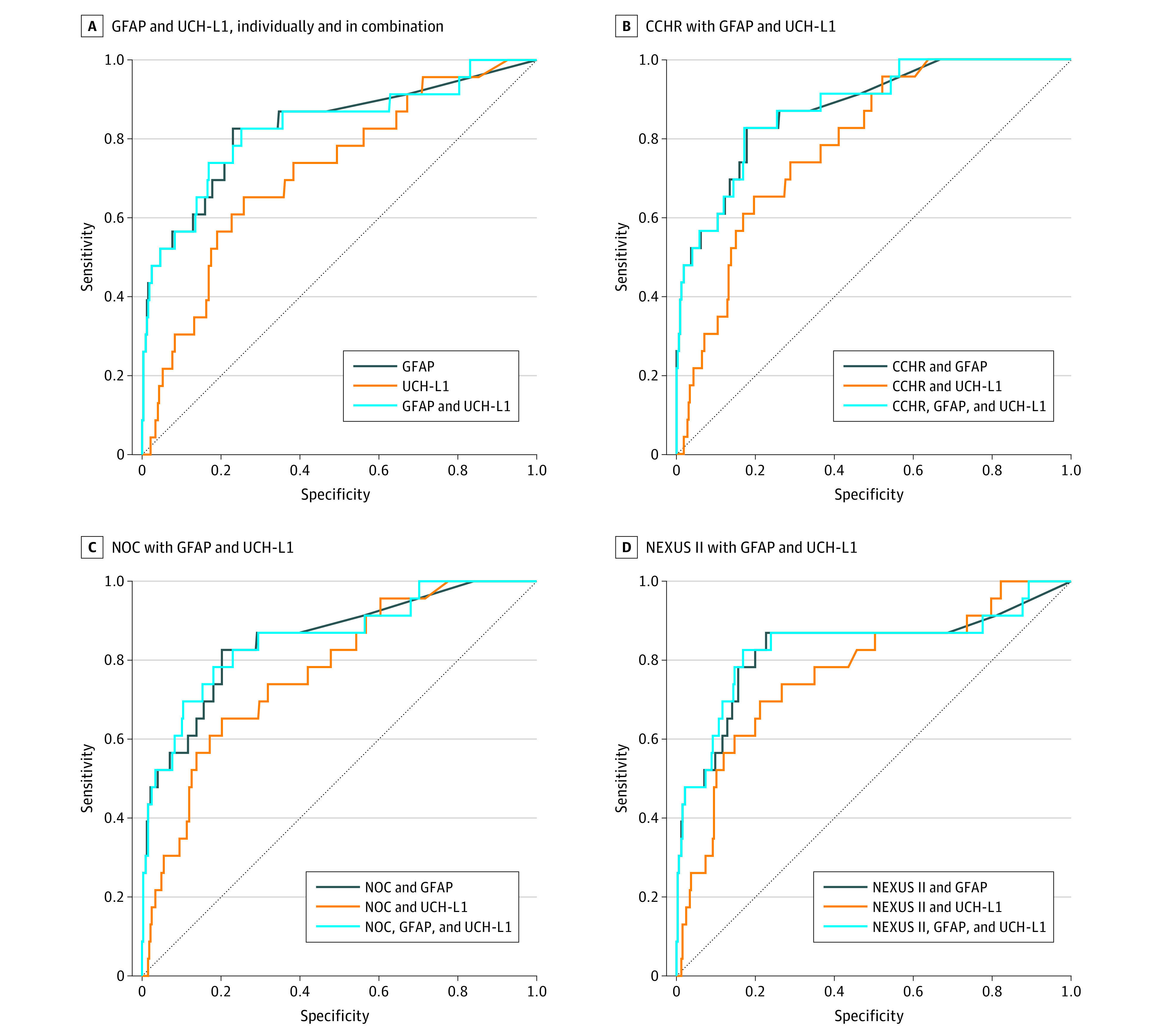
Comparison of the Area Under the Receiver Operating Characteristic Curves for Different Combinations of Biomarkers and Clinical Decision Rules in Different Combinations Sensitivity and specificity were measured for the presence of traumatic intracranial lesions detected on a computed tomography (CT) scan of the head. Gray dotted lines represent an AUROC of 0.5 and indicate no diagnostic discrimination. CCHR indicates Canadian CT Head Rule; GFAP, glial fibrillary acidic protein; NEXUS II, National Emergency X-Radiography Utilization Study II; NOC, New Orleans Criteria; and UCH-L1, ubiquitin C-terminal hydrolase.

The results of combining quantitative biomarker concentrations (range of values) with the clinical decision rules are shown in Figure 2. Measurement of GFAP alone (AUROC, 0.83 [95% CI, 0.73-0.93]) outperformed UCH-L1 (AUROC, 0.72 [95% CI, 0.61-0.82]) and was equivalent to GFAP plus UCH-L1 (AUROC, 0.83 [95% CI, 0.73-0.93]) (Figure 2A). The combination yielding the highest AUROC was CCHR plus GFAP (AUROC, 0.88 [95% CI, 0.81-0.95]), followed by NOC plus GFAP (AUROC, 0.85 [95% CI, 0.77-0.94]). Although the addition of UCH-L1 improved the performance of the clinical decision rules, it did not contribute as much as the addition of GFAP (AUROCs, 0.77-0.79) (Figure 2B-D). The combination of GFAP and NEXUS II was no better than GFAP alone (AUROC, 0.83 [95% CI, 0.72-0.94]) (Figure 2D).

A comparison of how comfortable the ED physicians were in applying each of the 3 decision rules clinically in patients with suspected MTBI is shown in eTable 3 in the Supplement. Most physicians felt very comfortable or comfortable with each of the 3 rules, including 211 of 346 respondents (61%) for the CCHR, 204 of 344 (59%) for the NOC, and 192 of 345 (56%) for NEXUS II. Fewer physicians felt uncomfortable or very uncomfortable with the rules, with 86 of 345 respondents (25%) for NEXUS II, 52 of 346 (15%) for the CCHR, and 47 of 344 (14%) for the NOC. When asked how regularly they used each of the rules, physicians listed NEXUS II most often (166 of 341 [49%]), followed by the CCHR (89 of 338 [26%]) and the NOC (54 of 324 [17%]). Interestingly, when physicians were asked “Would a biomarker blood test be useful in deciding to get a CT scan for this patient?” 83 (48%) responded yes, 68 (38%) responded maybe, and only 23 (13%) responded no. Therefore, 86% would consider a blood test useful compared with the 56% to 61% comfort levels with the decision rules.

## Discussion

This prospective cohort study conducted at a level I trauma center in the US is, to our knowledge, among the first to evaluate validated clinical decision rules, completed in real time by ED physicians during their clinical evaluations, against newly FDA-approved serum biomarkers GFAP and UCH-L1 in patients with suspected MTBI to detect intracranial lesions on CT scans. The sensitivities and specificities of the decision rules in this study were consistent with those of previously published studies,^8,24,25^ as were those of the biomarkers.^12,13,14,15,16,17,18^ The CCHR and NOC decision rules and GFAP plus UCH-L1 biomarkers had high sensitivities; the CCHR had the highest specificity and the greatest potential for reducing the use of CT. There was variability in the performance of GFAP and UCH-L1 levels based on the cutoff values used for the qualitative test. When quantitative biomarker concentrations were used, both biomarkers improved the performance of the clinical decision rules, but the combination of GFAP and UCH-L1 was not better than GFAP alone. Together, the CCHR and GFAP yielded the highest AUROC (0.88).

The TBI biomarker test that was approved by the FDA^23^ in 2018 uses prespecified cutoff values for both GFAP (22 pg/mL) and UCH-L1 (327 pg/mL) levels to provide a single dichotomous (qualitative) result (indicates yes or no) to determine whether a CT is necessary, with a sensitivity and specificity of 97% and 37%, respectively.^18^ Using this cutoff value and a second cutoff value from previous work (67 pg/mL for GFAP and 189 pg/mL for UCH-L1),^14,21^ important differences in the sensitivity (100% vs 91%) and specificity (25% vs 20%) were found, demonstrating that patients close to the threshold could be recategorized by slight changes in values. Moreover, patients with values close to the threshold could be categorized similarly to those who have results either far above or below the cutoff, although the severity of their injuries may be quite different. These biomarkers are known to have a graded response to injury (their concentrations increase as the injury severity increases),^12,13,14,15,16,17,18,19^ supporting the development of a quantitative test (using a range of values) instead of a qualitative test.

In fact, when the biomarkers were treated as continuous variables and combined with the clinical decision rules, the biomarkers improved the diagnostic performance of all 3 decision rules, with GFAP alone (without UCH-L1) contributing the most improvement. Even the addition of UCH-L1 did not improve performance of GFAP. This finding is supported by results in studies that show that GFAP and UCH-L1 together do not outperform GFAP alone.^15,19,26,27^ Even in the validation report that informed the FDA approval,^18^ a sensitivity analysis comparing the diagnostic accuracy of the biomarkers together and separately showed GFAP alone performed as well as GFAP and UCH-L1 together.

Although GFAP and UCH-L1 are FDA approved as a qualitative (yes or no) test, there is still much work to be considered before introducing them into clinical practice. For instance, the test does not use whole blood and requires blood to be processed (approximately 1 hour) before being measured and the purchase of specialized equipment not normally found in hospital laboratories. Moreover, in an editorial published in response to the biomarker validation study,^18^ the authors question whether 2 biomarkers (GFAP and UCH-L1) are really better than 1 (GFAP).^28^ They suggest that the choice of using 2 biomarkers was commercially driven because 2-biomarker assays can generate more income than a single-biomarker assay. Larger prospective independent studies will be required to answer these questions and should compare assay platforms, individual biomarker performance, and quantitative biomarker results.

One of the major strengths of this study is that the clinical factors from the 3 clinical decision rules were collected by the treating physicians during patient assessments and not extracted retrospectively by data extractors. Unfortunately, many studies extract clinical information from the medical record, introducing bias and inaccuracies through missing data, recording errors, misinterpretation, and lack of clinical judgment.^29^ Clinical decision rules require clinical interpretation that would be impossible for an extractor to determine, such as whether a clinician suspected a skull fracture, whether amnesia was antegrade or retrograde, or whether the clinician would have categorized the mechanism of injury as dangerous or trauma above the clavicle as significant. Optimally, clinical criteria should be completed by clinicians at the time of assessment.

Although clinical decision rules for MTBI have undergone multiple validation studies in the US and internationally, they have shortcomings.^30^ As evidenced by our physician survey, the rules are inconsistently applied among physicians, with some depending only on clinical judgment or on a combination of clinical judgment and select criteria from the decision rules.^30^ Some of the criteria listed in the decision rules are prone to interpretation (mechanism severity and physical examination findings), especially when a clear history of the event is not available or when a patient cannot adequately express their symptoms. The rules have shown variability in their sensitivities and specificities, with some missing intracranial lesions in exchange for reducing scans, and others leading to liberal scanning practices.^30^ As a result, having an objective measure, such as a blood test, is an attractive option.

Emergency physicians’ comfort levels with the decision rules were also collected in real time with each patient assessment and revealed that physicians had similar comfort levels across the 3 decision rules (56%-61%) but favored a blood test (86%). This could be a manifestation of practice patterns at this level I trauma center or physician apprehension about relying on decision rules instead of an objective blood test, perhaps owing to medicolegal concerns, criteria interpretation, or barriers beyond the scope of this discussion.

### Limitations

We recognize that there are limitations to this study. The study did not describe long-term outcomes or use high-resolution neuroimaging modalities such as magnetic resonance imaging to find subtle lesions that may be missed on CT imaging. However, the main outcome (lesions on CT) reflects current standards of practice.

Because this study was at a single trauma center, the generalizability to other centers may be limited, particularly to community hospitals. Nonetheless, this trauma setting is typical of many trauma centers around the country with an array of emergency and trauma physicians trained in different parts of the country. The demographics of our ED patient population are quite diverse and reflect a mixture of urban and suburban communities as seen in the racial and ethnic distribution of patients (Table 1). Although it is a tertiary care center, the hospital also acts as a community hospital, and patients are frequently transferred from rural communities for treatment.

Changes in cutoff values of GFAP and UCH-L1 yielded different results. The GFAP and UCH-L1 assay results used in this study and the validation study for FDA submission were both performed by the same biomarker company (Banyan Biomarkers, Inc) using the company’s own assays. Although the biomarkers were produced by the same company, the recommended cutoffs informing the FDA submission (22 pg/mL for GFAP levels and 327 pg/mL for UCH-L1 levels)^18^ were determined using chemiluminescence, whereas our study used sandwich ELISAs. This could explain the discrepancies in cutoff performance. Furthermore, the timing of sample collection differed between studies, with our study collecting samples within 4 hours of injury vs 12 hours in the validation study. Although the peak GFAP level is not reached until approximately 20 hours, the AUROC is still very good in detecting intracranial lesions on CT within 4 hours of injury with an AUROC of 0.84 at 4 hours and 0.82 at 12 hours.^15^ For UCH-L1 level, the peak is at 8 hours but the optimal time to detect intracranial lesions is within 4 hours of injury.^15^ It is crucial that these cutoffs be evaluated more carefully to ensure accurate classification of patients. As with the use of troponin levels to detect myocardial ischemia, it would be more informative for clinicians to have a quantitative TBI biomarker result (range of values) rather than a dichotomous qualitative one (yes or no).

Forty-six percent of eligible patients declined to participate, but patient characteristics were similar among enrolled and nonenrolled patients. Although a sample size was adequate for the primary outcome, the number of patients with outcomes of interest was limited to 23 (7%) and resulted in wide 95% CIs around the performance measures. Cohorts with MTBI and a larger number of CTs with positive findings will be needed to narrow the 95% CIs. On a positive note, the low incidence of intracranial injuries in the study population indicates that the sample represented a true population with MTBI and reflects the population for whom these decision rules were intended. It also reflects the population that would benefit most from selective use of CT scans.

## Conclusions

The findings of this cohort study suggest that although the CCHR, the NOC, and GFAP and UCH-L1 had equally high sensitivities, the CCHR had the highest specificity and the greatest potential for reducing the use of CT. The sensitivity and specificity of a qualitative GFAP-UCH-L1 test varied by the cutoff value used, but quantitative values enhanced the performance of the decision rules, with GFAP improving sensitivity and specificity independently of UCH-L1. The combination of the CCHR and GFAP had the best performance for detecting traumatic intracranial lesions on CT. Given these results, and the favorable view emergency physicians had of a blood test for MTBI, the addition of biomarker measurements to clinical decision rules has the potential to enhance clinical decision-making and warrants further study.

## Notes

Supplement. **eTable 1.** Proportion of Variables in Each Clinical Decision Rule Among Enrolled Patients **eTable 2.** Comparison of the AUROCs for Biomarkers and Clinical Decision Rules Independently Associated With Traumatic Intracranial Lesions on CT Scan of the Head **eTable 3.** Level of ED Physician Comfort With Each Decision Rule and the Biomarkers
