# Supplementary material for: Evaluation of Glial and Neuronal Blood Biomarkers Compared With Clinical Decision Rules in Assessing the Need for Computed Tomography in Patients With Mild Traumatic Brain Injury
Source: JAMA Netw Open. 2022 Mar 14;5(3):e221302. doi: 10.1001/jamanetworkopen.2022.1302 (PMC9907341; doi:10.1001/jamanetworkopen.2022.1302)
Supplement: Supplement. — eTable 1. Proportion of Variables in Each Clinical Decision Rule Among Enrolled Patients eTable 2. Comparison of the AUROCs for Biomarkers and Clinical Decision Rules Independently Associated With Traumatic Intracranial Lesions on CT Scan of the Head eTable 3. Level of ED Physician Comfort With Each Decision Rule and the Biomarkers [file jamanetwopen-e221302-s001.pdf]

# Supplementary Online Content

Papa L, Ladde JG, O'Brien JF, et al. Evaluation of glial and neuronal blood biomarkers compared with clinical decision rules in assessing the need for computed tomography in patients with mild traumatic brain injury. *JAMA Netw Open*. 2022;5(3):e221302. doi:10.1001/jamanetworkopen.2022.1302

**eTable 1.** Proportion of Variables in Each Clinical Decision Rule Among Enrolled Patients

**eTable 2.** Comparison of the AUROCs for Biomarkers and Clinical Decision Rules Independently Associated With Traumatic Intracranial Lesions on CT Scan of the Head

**eTable 3.** Level of ED Physician Comfort With Each Decision Rule and the Biomarkers

This supplementary material has been provided by the authors to give readers additional information about their work.

© 2022 Papa L et al. *JAMA Network Open*.

**eTable 1.** Proportion of Variables in Each Clinical Decision Rule Among Enrolled Patients

| **Variables from each clinical decision rule** | **All Patients (n=349) (%)** |
|---|---|
| **CANADIAN CT HEAD RULE** | |
| **High Risk (Total)** | **71 (20)** |
| GCS score < 15 at 2 hours after injury | 19 (5) |
| Suspected open or depressed skull fracture | 14 (4) |
| Any sign of basal skull fracture | 5 (1) |
| Vomiting ≥ 2 episodes | 6 (1) |
| Age ≥ 65 years | 33 (10) |
| **Medium Risk (Total)** | **170 (49)** |
| Amnesia before impact ≥ 30 minutes | 51 (15) |
| Dangerous mechanism | 194 (56) |
| **Low Risk (Total)** | **108 (31)** |
| **CCHR Positive** | **241 (69)** |
| | |
| **NEW ORLEANS CRITERIA** | |
| GCS < 15 | 35 (10) |
| Drug or alcohol intoxication | 14 (4) |
| Headache | 181 (52) |
| Age > 60 years | 52 (15) |
| Vomiting | 9 (3) |
| Persistent anterograde amnesia | 26 (7) |
| Seizure | 0 (0) |
| Trauma above the clavicle | 188 (54) |
| **NOC Positive** | **297 (85)** |
| | |
| **NEXUS II HEAD CT CRITERIA** | |
| Evidence of significant skull fracture | 8 (2) |
| Scalp hematoma | 97 (28) |
| Age >= 65 years | 33 (10) |

© 2022 Papa L et al. *JAMA Network Open*.

| | |
|---|---|
| Neurologic deficit | 8 (2) |
| Altered level of alertness | 70 (20) |
| Coagulopathy | 8 (2) |
| Abnormal behavior | 29 (8) |
| Persistent Vomiting | 4 (1) |
| **NEXUS Positive** | **173 (50)** |

[a] Some patients have more than one characteristic
[b] Percentages are rounded and may not equal 100%

© 2022 Papa L et al. *JAMA Network Open*.

**eTable 2.** Comparison of the AUROCs for Biomarkers and Clinical Decision Rules Independently Associated With Traumatic Intracranial Lesions on CT Scan of the Head

| Variable | AUROC (95% CI) | *P* value |
| --- | --- | --- |
| GFAP level cutoff 67 pg/mL | 0.76 (0.67-0.85) | <.001 |
| UCH-L1 level cutoff 189 pg/mL | 0.62 (0.52-0.72) | .050 |
| GFAP level cutoff 67 pg/mL and UCH-L1 level cutoff 189 pg/mL | 0.62 (0.53-0.72) | .046 |
| | | |
| GFAP level cutoff 30 pg/mL | 0.62 (0.52-0.72) | .053 |
| UCH-L1 level cutoff 327 pg/mL | 0.61 (0.50-0.72) | .08 |
| GFAP level cutoff 30 pg/mL and UCH-L1 level cutoff 327 pg/mL | 0.56 (0.44-0.67) | .38 |
| | | |
| CCHR | 0.67 (0.58-0.75) | .008 |
| NOC | 0.58 (0.47-0.69) | .20 |
| NEXUS II | 0.67 (0.57-0.78) | .005 |

Abbreviations: AUROC, area under the receiver operating characteristic curve; CCHR, Canadian CT Head Rules; CT, computed tomography; GFAP, glial fibrillary acidic protein; NEXUS II, National Emergency X-Radiography Utilization Study II criteria; NOC, New Orleans Criteria; and UCH-L1, ubiquitin C-terminal hydrolase.

© 2022 Papa L et al. *JAMA Network Open*.

**eTable 3.** Level of ED Physician Comfort With Each Decision Rule and the Biomarkers

| | CCHR (n=346) (%) | NOC (n=344) (%) | NEXUS II (n=345) (%) |
|---|---|---|---|
| **How comfortable would you be in following this rule for this patient?** | | | |
| **Very comfortable** | 49 (14) | 46 (13) | 52 (15) |
| **Comfortable** | 162 (47) | 158 (46) | 140 (41) |
| **Neutral/Unsure** | 83 (24) | 93 (27) | 67 (19) |
| **Uncomfortable** | 31 (9) | 36 (11) | 68 (20) |
| **Very uncomfortable** | 21 (6) | 11 (3) | 18 (5) |
| | | | |
| | **CCHR (n=338)** | **NOC (n=324)** | **NEXUS II (n=341)** |
| **Do you use this rule on a regular basis when evaluating MTBI patients for a head CT?** | | | |
| **I use this rule regularly** | 89 (26) | 54 (16) | 166 (49) |

© 2022 Papa L et al. *JAMA Network Open*.
